# Travel- and Community-Based Transmission of Multidrug-Resistant *Shigella* *sonnei* Lineage among International Orthodox Jewish Communities

**DOI:** 10.3201/eid2209.151953

**Published:** 2016-09

**Authors:** Kate S. Baker, Timothy J. Dallman, Adi Behar, François-Xavier Weill, Malika Gouali, Jeremy Sobel, Maria Fookes, Lea Valinsky, Ohad Gal-Mor, Thomas R. Connor, Israel Nissan, Sophie Bertrand, Julian Parkhill, Claire Jenkins, Dani Cohen, Nicholas R. Thomson

**Affiliations:** University of Liverpool, Liverpool, UK (K.S. Baker);; Wellcome Trust Sanger Institute, Hinxton, UK (K.S. Baker, M. Fookes, J. Parkhill, N.R. Thomson);; Public Health England, London, UK (T.J. Dallman, C. Jenkins);; Kimron Veterinary Institute, Bet Dagan, Israel (A. Behar);; Institut Pasteur, Paris, France (F.X. Weill, M. Gouali);; Centers for Disease Control and Prevention, Atlanta, Georgia, USA (J. Sobel);; Ministry of Health, Tel Aviv, Israel (L. Valinsky, I. Nissan);; Tel Aviv University, Tel Aviv (O. Gal-Mor, D. Cohen); Cardiff University, Cardiff, UK (T.R. Connor);; Scientific Institute of Public Health, Brussels, Belgium (S. Bertrand);; The London School of Hygiene and Tropical Medicine, London (N.R. Thomson)

**Keywords:** Shigella sonnei, shigellosis, Enterobacteriaceae, bacteria, enteric infections, diarrhea, dysentery, gastroenteritis, antimicrobial resistance, Jewish orthodox, Israel, Europe, France, Belgium, United States, Canada

## Abstract

Shigellae are sensitive indicator species for studying trends in the international transmission of antimicrobial-resistant *Enterobacteriaceae*. Orthodox Jewish communities (OJCs) are a known risk group for shigellosis; *Shigella sonnei* is cyclically epidemic in OJCs in Israel, and sporadic outbreaks occur in OJCs elsewhere. We generated whole-genome sequences for 437 isolates of *S. sonnei* from OJCs and non-OJCs collected over 22 years in Europe (the United Kingdom, France, and Belgium), the United States, Canada, and Israel and analyzed these within a known global genomic context. Through phylogenetic and genomic analysis, we showed that strains from outbreaks in OJCs outside of Israel are distinct from strains in the general population and relate to a single multidrug-resistant sublineage of *S. sonnei* that prevails in Israel. Further Bayesian phylogenetic analysis showed that this strain emerged approximately 30 years ago, demonstrating the speed at which antimicrobial drug–resistant pathogens can spread widely through geographically dispersed, but internationally connected, communities.

## Abstract 2

Shigellosis trends in these communities provide insight into global movement of drug-resistant *Enterobacteriaceae*.

Antimicrobial-resistant (AMR) *Enterobacteriaceae* are recognized as a global public health threat (*1*,*2*). Understanding the emergence of these pathogens and tracking transmission across international borders is vital for informing public health surveillance, intervention, and management (*3*). *Shigella* spp. are *Enterobacteriaceae* that cause severe, acute diarrhea resulting in mortality rates second only to rotaviruses as known agents of diarrheal disease (*4*). Shigellae cause disease in both low- and high-income nations (*5*), and >10 organisms can initiate disease (*6*). Shigellae are also increasingly resistant to antimicrobial drugs (*7*–*10*). Because of the large global burden of shigellosis, the low infective dose, highly visible disease syndrome, and ability to acquire AMR, shigellae are a relevant and sensitive indicator species for studying trends in the global transmission and emergence of AMR enteric bacteria.

Of the recognized *Shigella* spp., the distribution of *S. sonnei* makes it particularly relevant for studying international transmission because it is the most commonly isolated species in middle- to high-income nations (*5*) and causes a substantial disease incidence in low-income nations; for example, 23.7% of all documented shigellosis cases causing moderate to severe diarrhea in children <5 years of age in Africa and Asia (*11*). Moreover, *S. sonnei* prevalence increases as nations develop economically (*12*–*15*). To examine the underlying processes of such broad epidemiologic phenomena over medium- to long-term scales in bacterial populations, robust, high-resolution molecular subtyping is used. Subtyping of *S. sonnei* by using whole-genome sequencing has defined a global population structure that is divided into 4 lineages; the third lineage, global III, disseminated globally after acquiring multidrug resistance (MDR) (*16*). This advanced subtyping and established global context was used to show that the rise of *S. sonnei* in Vietnam was attributable to the point introduction and subsequent expansion of a single sublineage in the 1980s (*17*), demonstrating the effectiveness of this approach for characterizing epidemiologic phenomena.

Similarly, assessing the global burden of a widespread pathogen such as *S. sonnei* calls for use of a patient group in which the effects of illness are international. One such risk group for *S. sonnei* is Orthodox Jewish communities (OJCs) (*5*). These communities are highly susceptible to shigellosis because of densely populated living conditions, high numbers of young children per family, and intracommunity transfer facilitated by large holiday gatherings (*18*–*20*). *S. sonnei* shigellosis is highly endemic to Israel; its incidence there since the early 1990s has primarily been driven by biennial epidemics within OJCs in Israel (primarily in the 0–4-year age group, in whom the incidence is ≈7 cases/1,000 population/y [*19*]). In addition to incidence in OJCs in Israel, outbreaks ranging in size from 27 culture-confirmed cases to >13,000 cases of *S. sonnei* shigellosis have been reported in OJCs in Europe and North America (*18*,*20*–*24*). These outbreaks are attributable to highly clonal organisms, determined by using pulsed-field gel electrophoresis (*20*,*22*,*24*) and, in the case of an outbreak in Belgium, linked to prevailing strains from Israel (*22*). Thus, characterizing the international connectivity of OJC-associated *S. sonnei* represents an opportunity to assess the effects of travel- and community-based associations on the transmission of AMR *Enterobacteriaceae*.

We generated whole-genome sequences from >400 clinical isolates of *S. sonnei* collected over 22 years from OJCs within Israel, OJCs outside of Israel, and non-OJCs in the United Kingdom. We then combined these data with the established genomic global context for *S. sonnei.* By analyzing phylogenetic relationships, we investigated the distinction of strains from OJC outbreaks from other locally circulating strains (i.e., among non-OJCs) and explored the possible epidemiologic relationship of outbreaks in OJCs outside of Israel and endemic shigellosis in Israel. We also sought to determine the relationship of these epidemiologic processes with AMR determinants in *S. sonnei*.

## Materials

**Figure 1:**
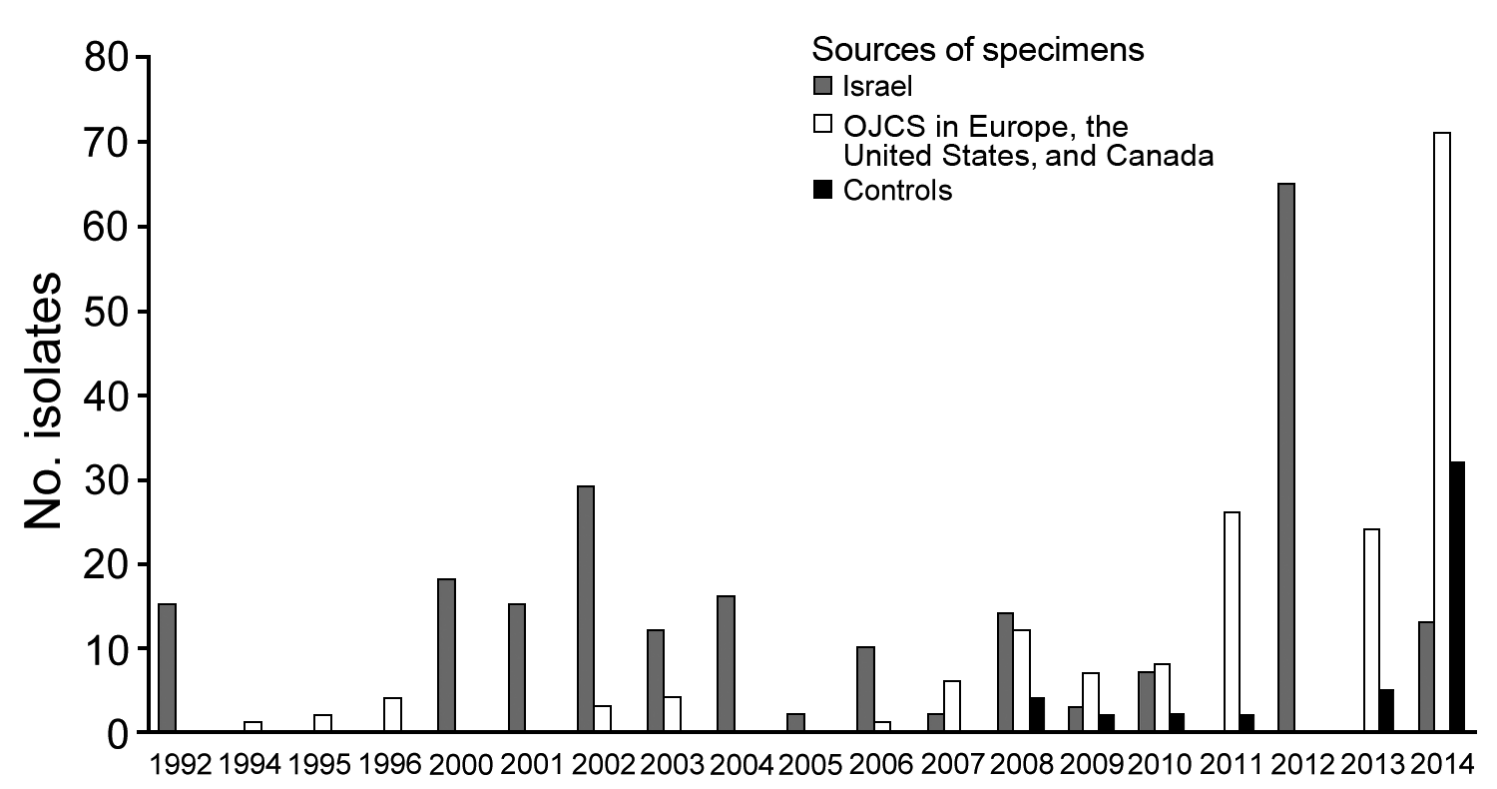
Origin and year of collection for 437 clinical isolates collected and sequenced from different countries and patient communities as part of study of travel- and community-based transmission of multidrug-resistant *Shigella* *sonnei* among international OJCs. Non-OJC samples were isolated from samples in the United Kingdom that were phage-type and temporally matched to isolates from OJCs in the United Kingdom (Technical Appendix 2 Figure 1). OJCs, Orthodox Jewish communities.

We performed whole-genome sequencing on 437 *S. sonnei* isolates as part of this study. These isolates were from patients associated with OJCs outside of Israel (n = 171), from 221 patients in Israel (200 OJC, 21 of unknown ethnicity), or from patients in the United Kingdom not associated with OJCs (n = 45) (Technical Appendix 1). The isolates were collected from 6 countries (Israel, the United Kingdom, France, Belgium, the United States, and Canada) during 1992–2014 (Figure 1). The collection included isolates from most previously reported OJC-associated outbreaks of *S. sonnei* shigellosis; we defined cases as being OJC-associated separately for each public health agency (Table 1).

**Table 1:** Origins of *Shigella sonnei* isolates used to track travel- and community-based transmission of multidrug-resistant *Shigella* *sonnei* among international Orthodox Jewish communities*

Region/community	Country	Year(s)	Details	References	No. isolates
Europe OJCs	Belgium	2008	Outbreak	(*22*)	3
	France	1996–2014	Multiple outbreaks	This study, (*21*)	64
	United Kingdom	2006–2014	Multiple outbreaks	This study, (*23*)	101
Europe non-OJCs	United Kingdom	2008–2014	Matched (time and phage-type) non-OJC cases	This study	45
United States and Canada OJCs	United States	1994–1995	Outbreak	(*24*)	3
Israel†	Israel	2000–2014	Sentinel laboratory surveillance	This study, (*19*)	221
Global context	Multiple	1943–2008	Used for background	(*16*)	118
Total					555
*OJC, Orthodox Jewish communities. †90% known OJC ethnicity.					

### Samples from Israel

The 221 samples of *S*. *sonnei* in Israel were collected during 1992–2014 in local hospital and health maintenance organization laboratories, including the national sentinel laboratory-based surveillance program (*19*). As described by Cohen et al. (*19*), 90% of the *S. sonnei* shigellosis isolates collected in Israel are from Jewish patients, and the isolates from Israel sequenced in this study were primarily (n = 200, 90%) derived from OJCs (Technical Appendix 2 Table 1).

### Samples from Outside of Israel

#### United Kingdom

A total of 146 *S. sonnei* samples were used from the Gastrointestinal Bacteria Reference Unit at Public Health England (London, United Kingdom). These samples included 22 from a small OJC-associated outbreak (*23*) and an additional 79 from outbreaks during 2006–2014 that were epidemiologically confirmed to be associated with OJCs by interviews and questionnaires as part of public health investigations. Also included were a set of 45 isolates from patients with no known OJC association (non-OJC). These background isolates were contemporaneously collected and selected on the basis of phage typing; that is, including diverse phage types, but focused on representing phage types associated with OJC outbreaks (Technical Appendix 2 Figure 1).

#### France

A total of 64 isolates from OJC-associated outbreaks in France (*21*) were submitted to the French National Reference Center for *E. coli, Shigella*, and *Salmonella* at the Pasteur Institute collected during 1996–2014. These isolates included those from a small OJC-associated outbreak in 2007 (*21*).

#### Belgium

Three *S. sonnei* isolates were provided from Belgium. These isolates were collected during a small OJC-associated outbreak in Antwerp in 2008 (*22*).

#### United States and Canada

Three representative isolates were collected during a large, homogenous, OJC-associated outbreak of *S. sonnei*. This outbreak occurred across the United States and Canada during 1994–1996 (*24*).

### Comparison Dataset

We analyzed the clinical isolates of *S. sonnei* alongside an existing global dataset compiled by KE Holt et al. (n = 118) (Technical Appendix 1) (*16*). In brief, this global context dataset comprises temporally (collected during 1943–2008, 70% collected after 1992) and geographically diverse (from 4 continents) *S. sonnei* isolates previously used to define the population structure of this pathogen. The dataset includes 1 sample collected in Israel in 2003.

## Methods

DNA was extracted at multiple sites by using the Wizard genomic DNA extraction kit (ProMega, Madison, WI, USA) according to manufacturer’s instructions. DNA was sequenced by using the MiSeq and HiSeq 2000 platforms (Illumina, San Diego, CA, USA) at multiple institutes according to in-house protocols (Technical Appendix 1) (*25*–*27*). Sequencing data for all isolates described passed internal quality control and were assembled into <687 contiguous sequences with a total length of <5.0 MB. Sequence data are available in the European Nucleotide Archive (http://www.ebi.ac.uk/ena; accession numbers in Technical Appendix 2).

Analysis of sequencing data was similar to that previously described (*28*). Multiple sequence alignment for phylogenetic analysis was generated by mapping to reference isolate *S. sonnei* Ss046 (GenBank accession no. CP000038), then masking mobile and repetitive elements (*16*) and stripping sites of recombination (*29*). Analysis of remaining variable sites was performed by using maximum-likelihood analysis in RAxML version 7.8.6 to create phylogenetic trees (*30*).

For isolates in the OJC-associated lineage for which the sampling date was known (n = 333; Technical Appendix 1), BEAST version 1.8 software (http://beast.bio.ed.ac.uk/) was used to estimate the emergence date of the lineage (*31*). Root-to-tip distances were generated by using Path-O-Gen version 1.4 (*32*). BEAST results shown are from 4 chains of 100 million Markov chain Monte Carlo generations run according to a general time reversible plus gamma substitution model, with a relaxed normal clock and Bayesian Skyline Population model, previously used for this pathogen (*16*,*17*). Chains were sampled every 1,000 generations with a 10% initial burn-in for root-height (time to most recent common ancestor) analysis. The maximum clade credibility tree was generated with a 10% burn-in and sampling every 100,000 generations. These results were consistent with those generated similarly by using a constant population growth model (Technical Appendix 2 Table).

De novo assembly, annotation, and antimicrobial resistance gene detection in the isolates was done as previously described (*28*) (accession numbers for annotated draft genome assemblies in Technical Appendix 1). Contiguous sequences containing antimicrobial resistance genes were extracted from assemblies and the presence of plasmid incompatibility groups on these contiguous sequences was determined by using PlasmidFinder (*33*). The presence of the Tn7/Int2 cassette was confirmed by mapping, and synteny detected by using ACT (*34*).

## Results

**Figure 2:**
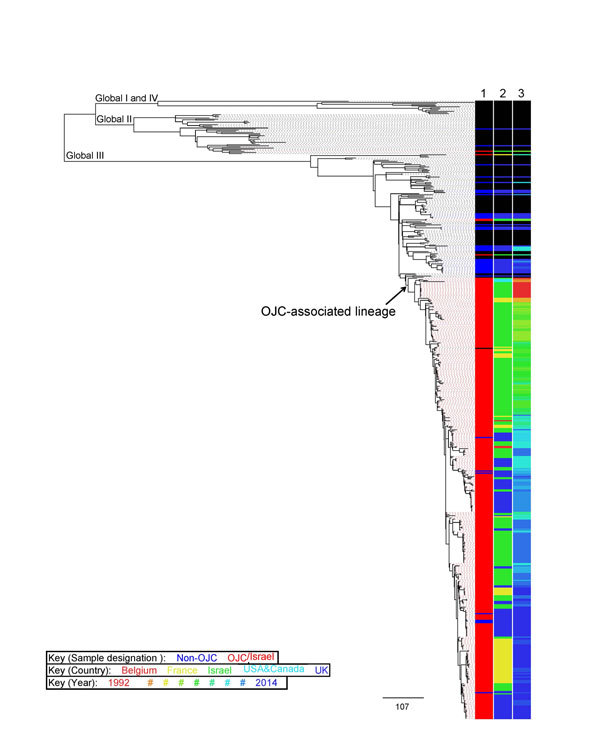
The OJC-associated lineage of multidrug-resistant *Shigella* *sonnei* in context with other global lineages. These background (non-OJC) isolates were contemporaneously collected and selected on the basis of phage typing; that is, including diverse phage types, but focused on representing phage types associated with OJC outbreaks. The midpoint rooted maximum-likelihood phylogenetic tree shows the relationships of 437 sequences from study of travel- and community-based transmission of multidrug-resistant *S.* *sonnei* among international OJCs compared with 118 isolates from a global context database of previously defined lineages of *S. sonnei*. Lineages are labeled along branches. Adjacent tracks are colored according to the inlaid key and show epidemiologic features of the isolate (lane 1, sample designation; lane 2, country; lane 3, year of isolation). Year of isolation is colored on a continuous heat map scale for 1992–2014, shown by hash symbols (#). Scale bar indicates single-nucleotide polymorphisms. OJCs, Orthodox Jewish communities.

To determine the relationships among *S. sonnei* from OJCs inside and outside of Israel, we constructed a phylogenetic tree including whole-genome sequence data from 437 isolates of *S. sonnei* alongside the 118 isolates from the global context dataset (Table 1; Figure 2). This analysis showed the existence of a large, unique monophyletic sublineage (n = 396 isolates) of the global III lineage that was almost exclusively (388/396, 98%) composed of isolates from OJC-associated outbreaks and samples from persons in Israel (OJC-associated lineage; Figure 2; Technical Appendix 2 Figure 2). This lineage contained nearly all isolates (217/221, 98%) sequenced from Israel and collected during1992–2014 (Figure 2); 170/171 (99%) of those identified from samples collected during the same time frame from OJCs in the United States, Canada, France, Belgium, and the United Kingdom; and the 1 isolate from the global context dataset that originated in Israel (Figure 2; Technical Appendix 1). The clustering of most (387/392, 99%) of the strains from Israel and the other OJC-associated strains in the OJC-associated lineage is remarkable considering that the lineage represented approximately 10% of the diversity of the *S. sonnei*: the largest intra-lineage pairwise distance was 8.8-fold less in the OJC-associated lineage relative to the remainder of the tree (Figure 2).

**Figure 3:**
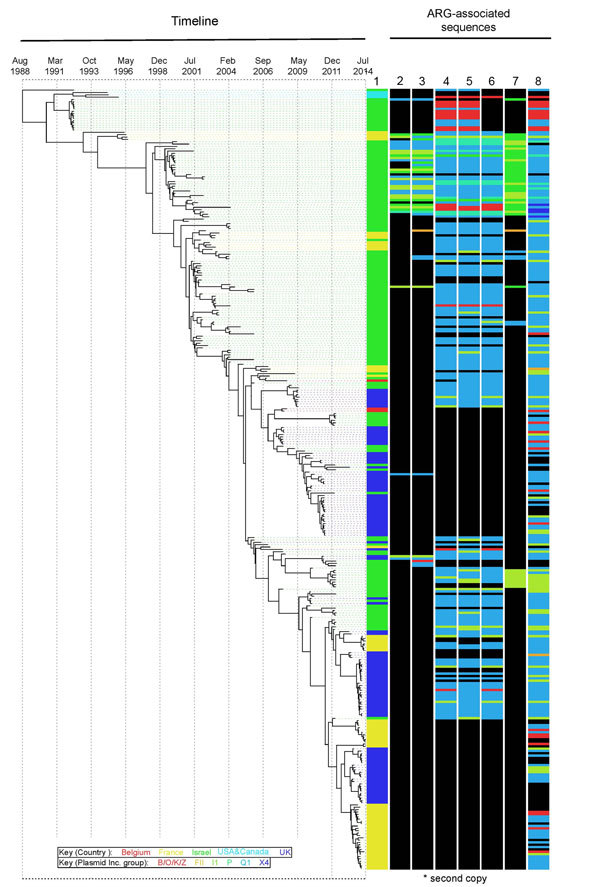
The OJC-associated lineage of multidrug-resistant *Shigella* *sonnei* across time and associated antimicrobial drug resistance. The Bayesian-inferred phylogenetic tree shows the evolutionary relationships of 333 isolates (those for which a fixed date was available) in the OJC-associated lineage since its emergence in the late 1980s. Tree tips overlay the collection date of the isolates. Lane 1 at right shows the country of origin (colors shown in key), and subsequent tracks show the plasmid incompatibility groups (colors shown in key) of contiguous sequences carrying the named antimicrobial resistance genes in that isolate: lane 2, *aadA1* (second copy); lane 3, *sul1*; lane 4, *strA*; lane 5, *strB*; lane 6, *sul2*; lane 7, *tetA*; lane 8, *bla*_TEM_. Black indicates gene not detected. The *aadA1* genes described in this figure are in addition to the copy carried by all isolates in the OJC-associated lineage on the Tn7/Int2 cassette described in Results. OJCs, Orthodox Jewish communities.

The inclusion of isolates from patients in the United Kingdom that were not associated with OJCs provided further illumination of the association of this lineage with OJCs. These non-OJC samples were almost entirely (37/45, 82%) located outside the OJC-associated lineage, distributed elsewhere in the global III and II lineages (Figure 2). Ultimately, this resulted in a statistically significant association of lineage with sample designation (i.e., OJC or non-OJC) among UK isolates (p<0.0001 by Fisher exact test). This association correlated better with phylogenetic position than phage type, which was a comparatively poor indicator of genome level phylogeny (Technical Appendix 2 Figure 3). The relative phylogenetic positions of non-OJC and OJC isolates from the United Kingdom when viewed in an international context, i.e., including the other strains (Figure 2) showed that strains from UK OJCs were more likely to be related to strains from Israel than to strains circulating in non-OJCs. For example, strains from OJCs sampled in the United Kingdom in 2014 were phylogenetically adjacent to strains from Israel sampled in 2014, rather than to non-OJC strains sampled in the United Kingdom in 2014 (Figure 2.)

Consistent with this finding, phylogenetic relationships within the OJC-associated lineage were defined more by time than geography (Figure 2; Technical Appendix 2 Figures 2, 4) Bayesian phylogenetic analysis showed that the OJC-associated lineage emerged in 1988 (95% highest posterior distribution 1985–1990) (Figure 3). Since that time, contemporaneously collected isolates were phylogenetically proximate with subsequent evolution, resulting in strain replacement rather than coexistence over time (Figure 3; Technical Appendix 2 Figure 2). Contrasting with the clear time signature in the lineage, geographic admixing of isolates occurred within the lineage (Figure 3). For example, >5 and 7 monophyletic clusters of isolates from OJC-associated outbreaks in France and the United Kingdom, respectively, were encompassed within the diversity of strains characterized in Israel (Figure 3). Samples from a single epidemiologic OJC-associated outbreak in Belgium were separated from each other by 44 single-nucleotide polymorphisms, making them phylogenetically distinct (Figure 2; Technical Appendix 2 Figure 2). This finding is consistent with contemporaneous OJC-associated outbreaks in different geographic areas representing real-time transmission events.

Because of the potential consequences of this intercontinental transmission to the transfer of AMR to *S*. *sonnei*, we determined the AMR characteristics of the OJC-associated lineage. We found that the lineage had a unique AMR profile relative to isolates outside of the OJC-associated lineage (Table 2). The OJC-associated lineage belonged to the global III lineage of *S. sonnei*, and every isolate in the OJC-associated lineage contained a Tn7/Int2 cassette that encoded the MDR thought to have facilitated the global dispersal of global III (*16*). This cassette contains the *aadA1, sat2*, and *dfrA1* genes that confer resistance to aminoglycosides, streptothricin, and trimethoprim and was chromosomally integrated adjacent to the *glmS* gene in these isolates (indicating a single acquisition event). This region is identical to a Tn7-like island, also adjacent to the *glmS* gene, in the newly emerging Xv serotype of *S. flexneri* (reference strain 2002017 [*35*]; Technical Appendix 2 Figure 5). No mutations known to confer quinolone resistance were found in *gyrA* or *parC* sequences of isolates in the OJC-associated lineage, and no plasmid-encoded quinolone resistance genes were detected (Technical Appendix 1).

**Table 2:** Antimicrobial resistance determinants among isolates isolates sequenced in study of travel- and community-based transmission of multidrug-resistant *Shigella* *sonnei* among international OJCs*

Antimicrobial resistance determinant	Within OJC-associated lineage, n = 395	Outside OJC-associated lineage, n = 42
*bl2d_oxa1*	0.00	0.02
*catA1*	0.00	0.02
*tetB*	0.00	0.05
*dfrA5*	0.00	0.07
*bl2b_tem*	0.00	0.00
*dfrA16*	0.00	0.00
*aac3iia*	0.01	0.00
*dfrA17*	0.01	0.00
*bl2b_tem1*	0.01	0.00
*mphA*	0.02	0.00
*dfrA14*	0.03	0.02
*sul1*	0.12	0.07
*tetA*	0.18	0.79
*strB*	0.53	0.88
*sul2*	0.53	0.90
*strA*	0.53	0.88
*arnA*	0.95	0.95
*mdtP*	0.97	1.00
*mdtO*	0.97	0.98
*mdtN*	0.97	1.00
*bacA*	0.99	0.98
*emrE*	0.99	1.00
*mdfA*	0.99	1.00
*mdtK*	0.99	1.00
*aadA1*	0.99	0.81
*macB*	0.99	0.98
*mdtL*	0.99	0.98
*mdtE*	0.99	1.00
*mdtF*	0.99	1.00
*mdtG*	1.00	1.00
*mdtH*	1.00	1.00
*dfrA1*	1.00	0.90
*acrA*	1.00	1.00
*acrB*	1.00	1.00
*bcr*	1.00	1.00
*bl1_ec*	1.00	1.00
*ksgA*	1.00	1.00
*tolC*	1.00	1.00
*bla*_TEM_†	0.86	0.14

*Excludes global context isolates. OJC-associated lineage defined in Technical Appendix Figure 1 (http://wwwnc.cdc.gov/EID/22/9/15-1953-Techapp1.pdf). OJC, Orthodox Jewish communities. †Detected separately.

To consider AMR mechanisms that had potential to mobilize among bacteria, we further examined antimicrobial resistance genes that were inconsistently present (in 5%–95% of isolates) (Figure 3; Technical Appendix 1; Technical Appendix 2 Figure 2). The 7 genes found to be inconsistently present across the lineage were second copies of *aadA1* present in some isolates; the aminoglycoside resistance–conferring *strA* and *strB* genes; the sulphonamide resistance genes *sul1* and *sul2*; the tetracycline resistance gene *tetA*; and the ampicillin resistance gene *bla*_TEM_ (Figure 3; Technical Appendix 2 Figure 2). Attempts were made to determine the coinheritance and genetic carriage elements of these genes (within the limitations of genome assembly). In isolates that had additional copies of *aadA1,* the gene was typically co-inherited with the *sul1* gene (Figure 3; Technical Appendix 1; Technical Appendix 2 Figure 2); this combination was found on plasmids of 2 different incompatibility groups, I1 and P, as well as on contiguous sequences where no plasmid incompatibility groups were identified, shown as unknown (Figure 3; Technical Appendix 2 Figure 2). Similarly, *strA*, *strB*, and *sul2* were frequently co-inherited and found on plasmids of 4 different incompatibility groups. Isolates collected earlier in the lineage’s evolution tended to carry *strA/strB/sul2* on B/O/K/Z, Q1, and P incompatibility group plasmids, whereas later isolates carried the genes on I1 plasmids (Figure 3; Technical Appendix 2 Figure 2). Similarly, the *tetA* gene appeared to have had 2 major introductions into the lineage, earlier on a P group plasmid and later on an I1 plasmid (Figure 3; Technical Appendix 2 Figure 2). Last, *bla*_TEM_ genes were found in 86% of isolates in the lineage, compared with 14% outside of the lineage (Table 2; Technical Appendix 1); these genes were carried on plasmids of 5 different incompatibility groups, with sporadic coinheritance patterns with other resistance genes (Figure 3; Technical Appendix 2 Figure 2).

## Discussion

We used whole-genome sequencing to develop a high-resolution picture of the international transmission of *S. sonnei* and its AMR determinants among OJCs over several decades. These analyses offer insight for the epidemiology of shigellosis inside and outside of Israel as well as for the broader transmission of AMR enteric pathogens. We showed that, in countries outside of Israel, outbreak strains in OJCs were distinct from strains circulating in the general population and that OJC-associated strains were more closely affiliated with outbreaks associated with OJCs in other countries (irrespective of geographic distance) and strains circulating in Israel. Strains from Israel and strains from nearly all previously reported OJC outbreaks elsewhere formed a distinct OJC-associated sublineage that emerged ≈30 years ago. Unlike other described emergent *Shigella* sublineages (*17*,*28*), the OJC-associated lineage lacked a defining association with AMR.

Isolates collected during outbreaks of *S. sonnei* in OJCs outside Israel were phylogenetically linked to contemporaneous isolates from Israel. This finding was suspected from previous studies that used pulsed-field gel electrophoresis, the results of which supported that samples from outbreaks among OJCs in the United States and Belgium were distinct from samples of *S. sonnei* circulating locally in non-OJCs (*22*,*24*) and were related to strains from Israel (*22*). We confirmed this link in an analysis of specimens collected in the United Kingdom that showed that strains from OJC-associated outbreaks were distinct from other circulating strains in that country but related to contemporaneous strains from Israel (Figure 2). The broader analysis, expanded in time and geography, showed that local epidemics in OJCs in France, Belgium, and North America (*21*,*22*,*24*) were also linked with contemporaneous isolates from Israel (Figure 3; Technical Appendix 2 Figure 2). This pattern of phylogenetic clustering by community affiliation was also recently demonstrated for *S. flexneri* 3a strain transmission among a global epidemiologic community of men who have sex with men, through which a unique MDR sublineage spread during ≈20 years (*28*). These studies demonstrate the speed with which AMR *Enterobacteriaceae* can be transmitted among persons in an internationally linked community rather than by contiguous geographic spread.

These findings also have implications for the epidemiology of shigellosis within Israel. The isolates from Israel in this study derive primarily from OJCs, which drive cyclic *S. sonnei* epidemics in Israel (*19*); here, they were shown to belong to a single, low-diversity sublineage. The lineage was monophyletic and had a strong time signature, consistent with a point introduction and subsequent epidemic emergence. This pattern was similar to that observed in Vietnam after the introduction of another global III *S. sonnei* sublineage (*15*,*17*). The date of the emergence of the OJC-associated lineage (1988 [95% highest posterior distribution 1985–1990]) is consistent with that estimated in a previous study where a 2-isolate lineage emerged in the Middle East during 1983 (*16*). Considering the timing and context of the emergence, it is possible that the OJC-associated lineage emerged from the large waterborne epidemic that occurred in Israel in the mid-1980s (*19*) or was potentially introduced from the first reported OJC-associated outbreak of shigellosis outside of Israel, which was an outbreak of >13,000 cases across the United States that also occurred in the 1980s (*18*). Samples from this period were not available to explore these origins of these outbreaks, but it is clear that the OJC-associated lineage is now endemic to OJCs in Israel and is causative of OJC-associated outbreaks elsewhere.

The AMR profile of the OJC-associated lineage is consistent with the phenotypic information and is likely influenced by antimicrobial resistance selection pressures in the 0–4-year age group, which is primarily affected by shigellosis in OJCs. Sulfonamide and tetracycline resistance determinants were in flux across the lineage (Figure 3), and these antimicrobial classes have been reported as being phenotypically dynamic over time among *S. sonnei* isolated in Israel (*19*). Furthermore, trimethoprim resistance was chromosomally encoded in all isolates, and plasmid-mediated ampicillin resistance was a common finding (86% of isolates) (Figure 3). These antimicrobial classes are key for the treatment of children with shigellosis. Similarly, resistance to tetracyclines, which can be used in children >7 years of age (*8*), and macrolides (*mphA* gene [*21*]) (Table 2) were also found. Despite being reported in other global III *S. sonnei* strains (*16**,**17*), resistance to quinolones was not found in the OJC-associated lineage, possibly because the use of quinolones is contraindicated in children.

The acquisition of the Tn7/Int2-encoded MDR in this lineage may have facilitated its epidemic emergence, as has been hypothesized for the broader global III lineage (*16*) and as is possible for *S. flexneri* Xv (*35*), although these possibilities cannot be explored fully by using these data. The presence of this gene cassette throughout the lineage and other phylogenetic clusters of antimicrobial resistance genes demonstrates that AMR can spread through this geographically dispersed, but closely associated, community. However, in contrast to the emergence of *S. flexneri* 3a among men who have sex with men or of *S. sonnei* in Vietnam, further acquisition of AMR does not appear to have shaped the subsequent evolution of the lineage. The presence of additional resistance genes (Figure 3) was not correlated with later time points, and the same genes were carried on distinct mobile genetic elements, consistent with sporadic reintroduction, rather than maintenance of the additional resistance genes in the population. This absence of a defining association with AMR suggests that it is probably primarily the epidemiologic suitability of OJCs to the transmission of *S. sonnei* that supports its maintenance in these communities. This likelihood is consistent with previous studies of OJC-associated shigellosis, which suggest that transmission is largely driven by communal childcare arrangements in a host population that is young and densely structured (*19*,*20*,*22*).

This study documents the speed with which MDR enteric bacteria can transmit intercontinentally through travel within a geographically dispersed, but closely linked, community. Awareness of this mode of sustained, geographically noncontiguous transmission must inform public health practice, including targeted control and reduction of consequences of the pathogen through developing effective relationships with affected communities; identifying specific risk factors; and designing, piloting, and eventually implementing specific culturally appropriate interventions with the participation and support of the community while avoiding stigmatization. Effective ongoing surveillance is also vital, and we demonstrated the resolution required for that purpose and the need for effective data sharing to track these otherwise silent transmission phenomena. Furthermore, the repeated application of high-resolution tools supports identification of parallels and contrasts with previous genomic epidemiology studies, supporting construction of a complete picture of the global transmission of AMR enteric pathogens, advancing our position for tackling this global public health issue.

Technical Appendix 1 Sample details for isolates of *Shigella* *sonnei* used in this study and reference strains from global context database.

Technical Appendix 2 Supporting data and phylogenetic and genetic characteristics of isolates of *Shigella* *sonnei*.
