# Supplementary material for: Travel- and Community-Based Transmission of Multidrug-Resistant Shigella sonnei Lineage among International Orthodox Jewish Communities
Source: Emerg Infect Dis. 2016 Sep;22(9):1545–53. doi: 10.3201/eid2209.151953 (PMC4994374; doi:10.3201/eid2209.151953)
Supplement: Technical Appendix 2 — Supporting data and phylogenetic and genetic characteristics of isolates of Shigella sonnei. [file 15-1953-Techapp-s2.pdf]

Article DOI: http://dx.doi.org/10.3201/eid2209.151953

Travel- and Community–Based Transmission of Multidrug-Resistant *Shigella sonnei* Lineage among International Orthodox Jewish Communities

Technical Appendix 2

Technical Appendix 2 Table. Mean and effective sample size of parameter values of multidrug resistant *Shigella sonnei* lineage among orthodox Jewish communities in Bayesian phylogenetic analysis*

	Bayesian skyline population		Constant population	
Parameter	Mean	ESS	Mean	ESS
Posterior	-4.69×10^{6}	792.807	-4.68×10^{6}	7,522.987
Prior	–5153.919	609.238	–2833.226	5,410.943
likelihood	-4.68×10^{6}	5387.264	-4.68×10^{6}	54,685.64
Tree model root height	9,370.712	306.475	9,771.741	6,421.851
ac	0.4	10,986.655	0.4	77,926.097
ag	1.001	9,183.302	1.002	65,332.289
at	0.379	11,166.892	0.379	79,525.799
cg	0.2	12,399.209	0.2	92,649.665
gt	0.434	10,380.523	0.434	79,933.061
Frequencies1	0.247	3495.52	0.247	27,482.92
Frequencies2	0.252	3,398.894	0.252	27,574.623
Frequencies3	0.254	3,282.012	0.254	25,521.903
Frequencies4	0.247	3,514.047	0.247	26,662.601
ucld.mean	5.57×10^{9}	474.286	5.433×10^{9}	7,102.002
ucld.stdev	0.511	1,431.808	0.522	6,809.351
Mean rate	5.33×10^{9}	399.931	$5{,}188 \times 10^{9}$	7,440.114
Coefficient of variation	0.546	1,587.367	0.557	14,508.14
Covariance	1.64×10^{2}	11,371.346	0.01437	89,859.262
Tree likelihood	-4.68×10^{6}	5,387.264	–4,681,000	54,685.64

*ESS, effective sample size; UCLD, uncorrelated lognormal distribution; stdev, standard deviation.

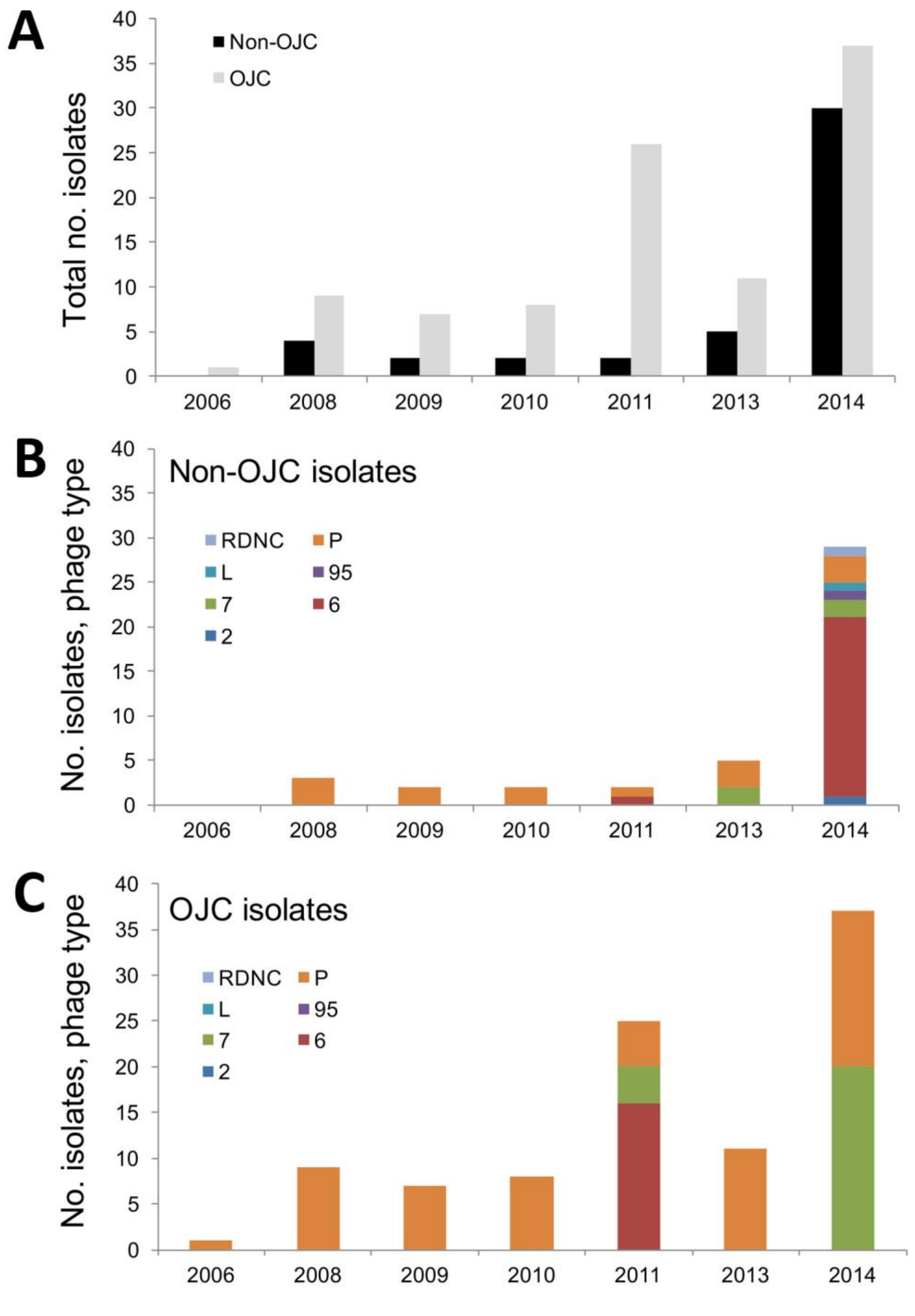

Technical Appendix 2 Figure 1. Clinical isolates collected from the UK as part of this study. The uppermost chart shows the breakdown of 146 isolates sequenced from UK by designation to Orthodox Jewish Communities (OJC, n=101) or non-Orthodox Jewish communities (non-OJC, n= 45) over time. The stacked charts below show the breakdown of these isolates by phage type over the same time scale.

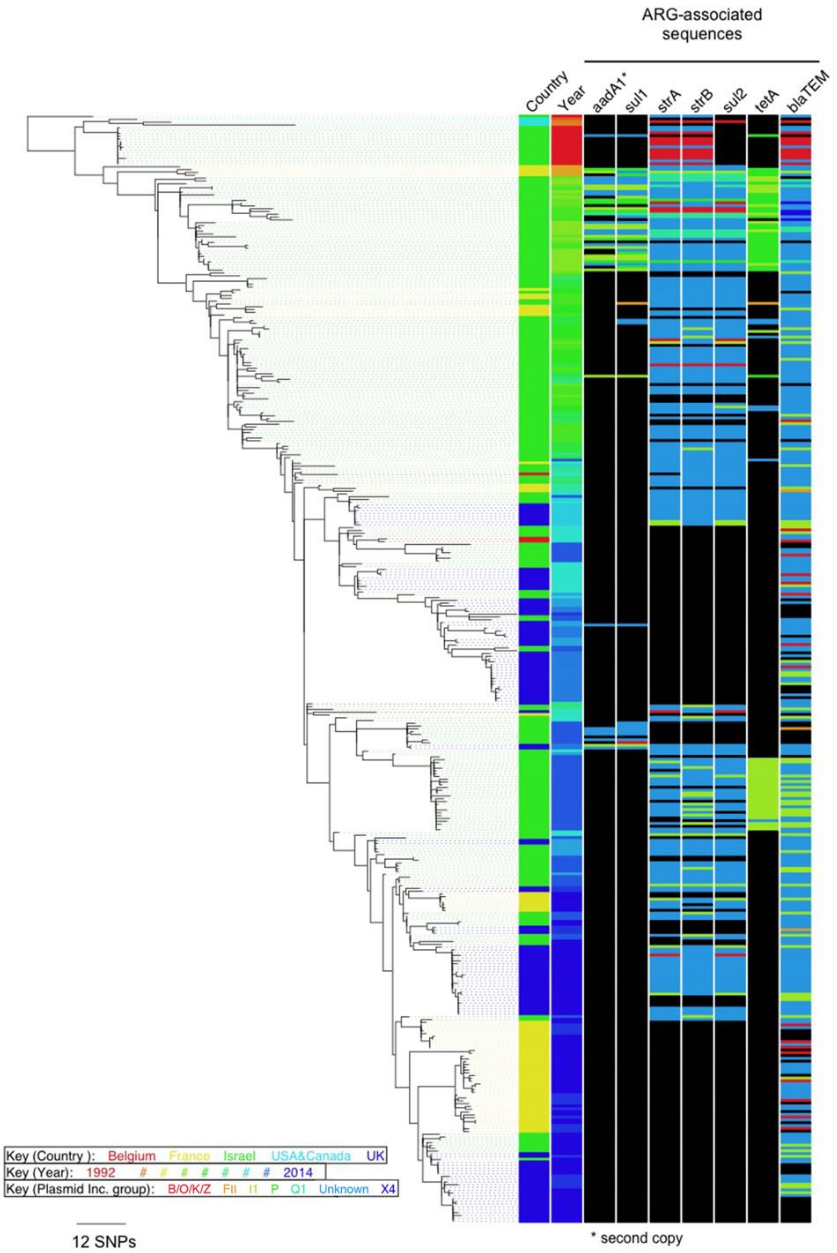

Technical Appendix 2 Figure 2. Increased resolution view of the OJC-associated lineage from Figure 2. The maximum-likelihood phylogenetic tree shows the evolutionary relationships of all 396 isolates in the OJC-associated lineage. The first adjacent colour track shows the country of origin coloured according to the inlaid key, and the second shows the year of isolation coloured on a continuous heat map scale from 1992 to 2014 shown by inlaid hash symbols. Subsequent tracks show the plasmid incompatibility (Inc.) groups of contiguous sequences carrying the named ARGs in that isolate according to the key below (a black colour in these columns indicates the ARG was not detected). The aadA1 genes described in this figure are in addition to the copy carried by all isolates in the OJC-associated lineage on the Tn7/Int2

cassette described in the results. ARG, antimicrobial resistance genes; OJC, Orthodox Jewish community; UK, United Kingdom; USA, United States of America.

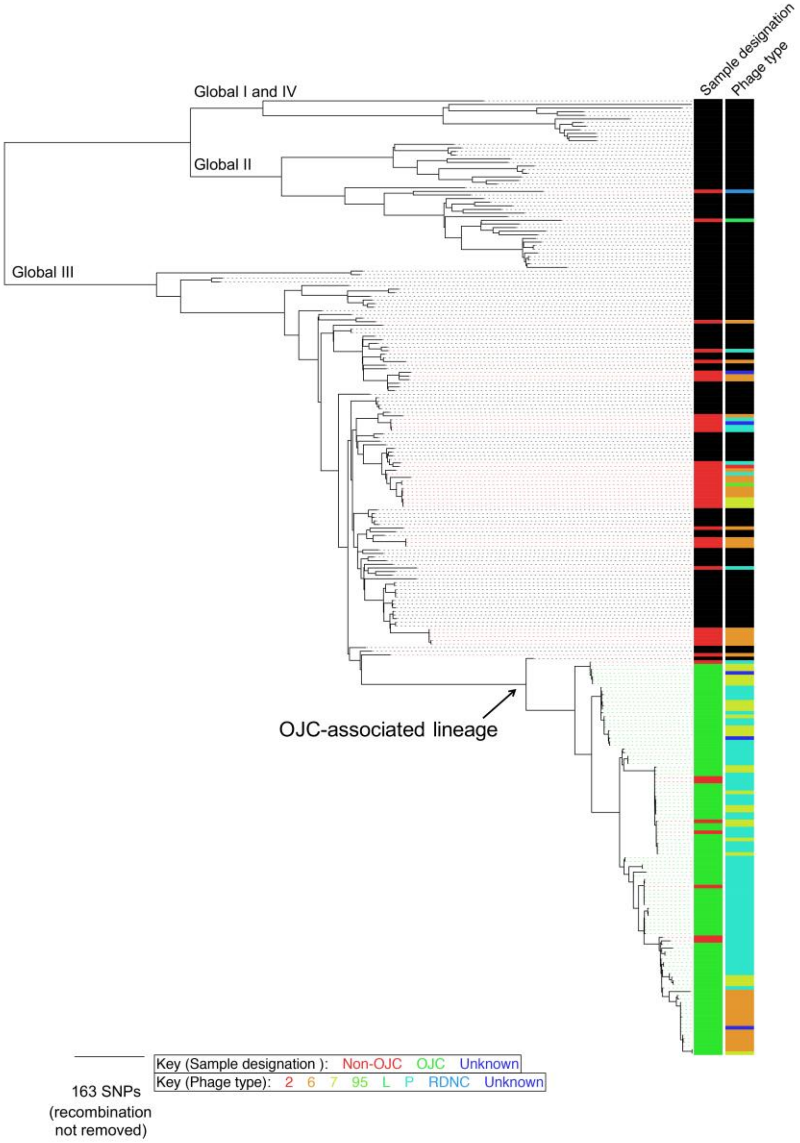

Technical Appendix 2 Figure 3. A national (United Kingdom) view of the OJC-associated lineage in context. The midpoint rooted maximum-likelihood phylogenetic tree shows relationships among UK isolates in this study (n = 146, collected during 2006–2014) within the previously defined lineages of the *S. sonnei* global context (n = 118, indicated by black in adjacent columns). Tracks adjacent to tree tips show the sample designation and isolate phage type colored according to the figure legend. The OJC-associated lineage is labeled with an arrow over the internal node, and recombination has not been removed. Scale bar indicates SNPs (single nucleotide polymorphisms). OJC, Orthodox Jewish communities.

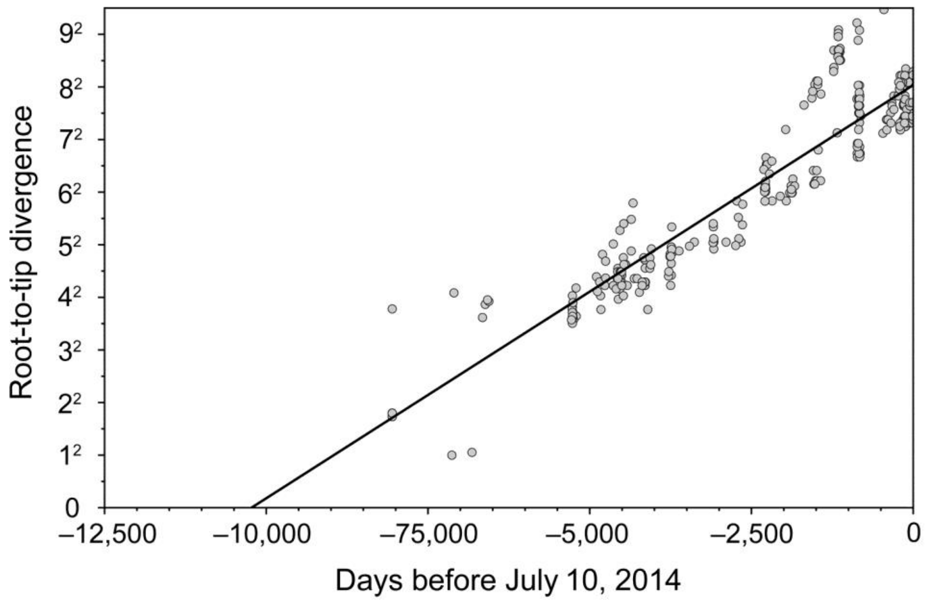

Technical Appendix 2 Figure 4. Root-to-tip divergence of the maximum likelihood tree in Figure S1 is shown as a function of time (isolation date in days before July 10, 2014).

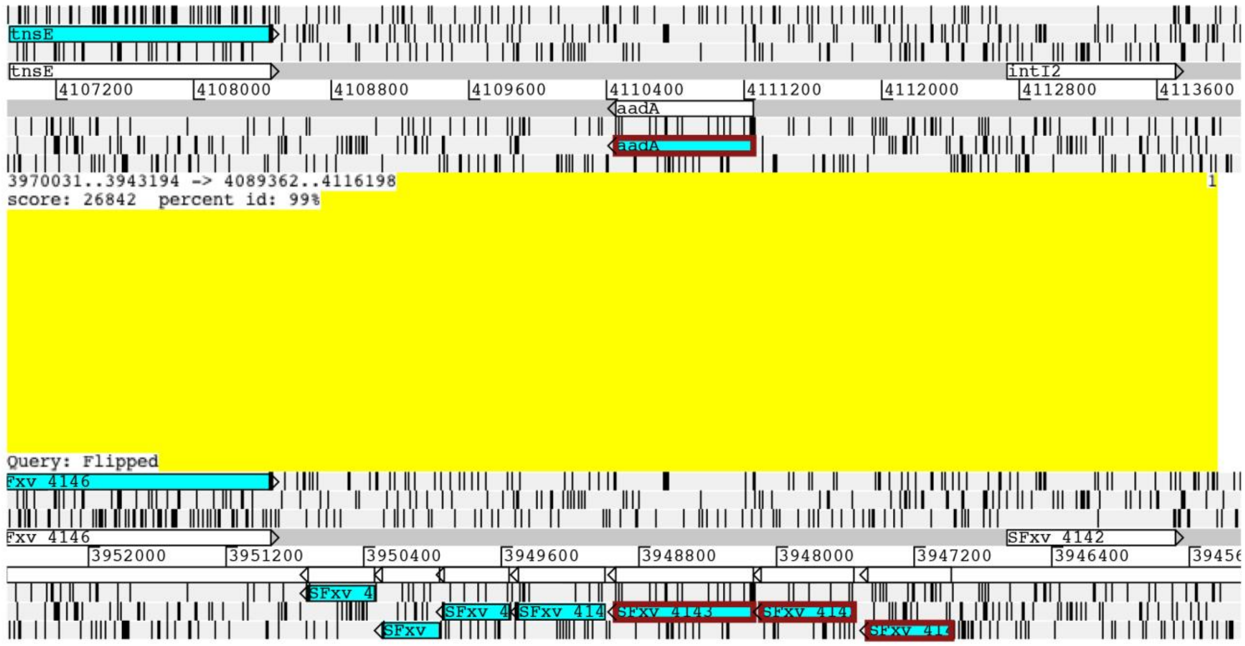

Technical Appendix 2 Figure 5. Synteny of the Tn7/Int2 cassette in *Shigella* reference genomes. Synteny defined by BLAST comparison of *S. sonnei* Ss046 (upper), isolated in the 1950s, and *S. flexneri* serotype Xv (lower) isolated in 2002, is shown in yellow. The orientation of *S. flexneri* has been flipped to show inverted synteny. The aadA1, sat2, and dfra1 genes are highlighted in maroon. The sat2 and dfrA1 genes are not annotated in the *S. sonnei* reference genome, but stop codons overlying each reading frame (vertical black marks) show the corresponding open reading frames.
